# The Current Practice of Adult Idiopathic Scoliosis Surgical Treatment Among Spine Surgeons in Saudi Arabia

**DOI:** 10.7759/cureus.11552

**Published:** 2020-11-18

**Authors:** Abdullah N Al-qahtani, Khalid S Al Aqeely, Zeyad Alamari, Sami Al Eissa, Fahad Alhelal, Ali A Alhandi, Faisal Konbaz

**Affiliations:** 1 Medicine, King Abdulaziz Medical City, Ministry of National Guard Health Affairs, Riyadh, SAU; 2 Orthopaedics, King Abdulaziz Medical City, Ministry of National Guard Health Affairs, Riyadh, SAU

**Keywords:** orthopedic, neurosurgery, spine, ais, saudi arabia, adolescent idiopathic scoliosis (ais), adult idiopathic scoliosis, scoliosis

## Abstract

Background: Scoliosis is a spinal deformity consisting of lateral curvature and rotation of the vertebrae. The goal of corrective scoliosis surgery is to restore alignment of the spine and maintain motion. However, there is no global agreement among spine surgeons on the best method of surgical intervention, and little is known about the variations in practice among spine surgeons in Saudi Arabia.

Objectives: The aim of this study is to examine the current surgical practices relating to adolescent idiopathic scoliosis and to establish the degree of variation and consensus among spine surgeons in Saudi Arabia.

Methods: This was a cross-sectional study conducted among spine surgeons in Saudi Arabia, using a self-administered questionnaire. The questionnaire addressed four areas: surgeons’ demographics, pre-operative assessment, operative assessment, and infection control practices. There were a total of 27 questions. Data was analyzed using statistical package for social sciences (SPSS) software (SPSS Inc., Chicago, IL, USA).

Results: A total of 150 surgeons were included in this study. Of these, 73 responded to the questionnaire (response rate: 48.6%), 46 (63.01%) of whom were fellows. All respondents were males, and 37 (51.39%) were aged between 30 and 40 years. Fifty five (75.34%) were orthopedic surgeons. The most commonly used pre-operative health assessment test was an echocardiogram. Sixty six (90%) surgeons requested scoliosis anteroposterior (AP) view and lateral radiograph for curvature between 40 degrees and 90 degrees, and 65 (89%) requested it for curvature greater than 90 degrees. For the posterior construct, 29 (45.31%) did not use cross-links for fewer than 10 levels, and 23 (35.94%) did not use cross-links for more than 10 levels. In addition to a local bone graft, the most commonly used transplant method was allograft 37 (50.68%). Fifty five (85.94%) respondents favored an all-screw pedicle construct over a hybrid construct.

Conclusion: A large variation in surgical practices was observed. This was especially apparent in practices including the use of cross-links, intra-operative cell salvage, and the intra-operative use of traction. Some degree of variation was observed in pre-operative imaging assessment practices. Surgical guidelines should be developed to build a consensus among surgeons. A unified surgical training curriculum may help in achieving this goal.

## Introduction

Scoliosis is a spinal deformity consisting of lateral curvature and rotation of the vertebrae [1]. Typically, this curve is "S"- or "C"-shaped over three dimensions [1,2]. In some cases, the degree of curvature is stable, while in others scoliosis can lead to a progressive deterioration in the curvature of the spine [3]. Usually, mild scoliosis does not cause problems, and patients do not complain of pain. In its more severe forms, however, it can interfere with breathing [3-5]. In most cases, the etiology of scoliosis is unknown. However, this is believed to be multifactorial and involve a combination of environmental and genetic factors [3]. Despite its unknown etiology, family history is considered to be one of the risk factors for scoliosis [1]. It can also be secondary to other diseases such as cerebral palsy, muscles spasms, and Marfan syndrome [1].

Diagnosis of scoliosis is confirmed by x-ray [1]. It is generally classified as either “functional,” where the underlying spine is normal, or “structural,” where the curve is fixed [1]. The goal of corrective scoliosis surgery is to restore alignment of the spine and to maintain motion. While there are differences in scoliosis surgery, good-quality evidence of best practices is scarce, as randomized clinical trials entail many ethical challenges [6].

Michael et al. have observed significant differences in scoliosis surgery in the United Kingdom [7]. This is despite efforts to standardize clinical practice and the publication by the British Orthopedic Association of clinical guidelines for scoliosis treatment [8]. Both clinical and surgical discrepancies were observed between spine surgeons [7]. Similarly, in a study from the United States, Sanders et al. gave scoliosis surgeons four different cases and asked about instrumentation use and fusion levels. The variation among respondents was significant in infusion level and instrumentation [9]. Likewise a four-country survey by Debono et al. addressed five degenerative spinal conditions, one of which was degenerative scoliosis in an 80-year-old female [10]. The survey showed a significant variation in surgical approach between spine surgeons and lack of literature consensus.

A survey by de Kleuver et al. of spine surgeons from different countries sought to obtain a consensus on optimal operative care for adult idiopathic scoliosis (AIS) patients [6]. Variations in surgical practice were observed in the absence of this consensus. Aubin et al. published similar findings; however, they hypothesized that these observed differences appear to be due to surgeons’ preference based on experience [11]. Daffner et al., meanwhile, conducted a retrospective database review of a large number of US AIS patients to identify variations in costs and surgical techniques [12]. The study found no significant cost differences between the various surgical techniques.

In the absence of holistic evidence-based guidelines, surgeons depend on their clinical judgment and experience. General guidelines and standardized practices exist for the timing of scoliosis operations. However, surgical practice is still not governed by any guidelines or algorithms.

Assessing the degree of variation is crucial for cost effectiveness, patient satisfaction, and positive outcomes. This is especially the case for local practices. Zygourakis et al. reported a substantial increase in the cost of spinal deformity surgery, including scoliosis, over the last decade in the United States [13]. The authors cited both patient and hospital factors as reasons for this increase. The study did not emphasize variations in surgical practices; however, such differences may contribute to these inflated costs.

To our knowledge, no variations in scoliosis surgical practice have been documented in Saudi Arabia. The aim of this study is hence to study current practice among spine surgeons and to establish the degrees of variation, differences, and consensus in the country.

## Materials and methods

This was a cross-sectional study using a self-administered survey. The aim was to explore variations in the surgical management of AIS patients by spine surgeons in Saudi Arabia. All spine surgeons currently working in Saudi Arabia were contacted, with contact details provided by the Saudi Spine Society’s Scientific Committee. The estimated number of spine surgeons in Saudi Arabia (either orthopedic or neurosurgery) was approximately 150 based on the database of Saudi Spine Society's Committee, with an anticipated response rate of 50%.

An electronic questionnaire was designed to ask spine surgeons attending the Saudi Spine Society annual meeting about the optimal treatment of AIS patients in Saudi Arabia. The content of the questionnaire was reviewed by two senior orthopedic surgeons, and it was distributed electronically using Survey Monkey (SurveyMonkey Inc., San Mateo, California, USA). To increase the response rate, spine surgeons who did not respond were provided with a hardcopy of the survey.

The survey addressed four different areas: surgeons’ demographics, pre-operative assessment, operative assessment, and infection control. There were a total of 27 multiple choice questions, with the option to choose more than one answer in some instances. The surgeons’ demographics section included eight questions relating to current title, gender, age, specialty, years of experience, number of scoliosis operations per year, sector of practice, and region of practice in Saudi Arabia.

The pre-operative section contained seven questions and two sub-sections: (1) pre-operative patient health assessment, including questions about the frequency of performing pulmonary function test (PFT), cardiac echocardiogram, nutritional blood tests, and pre-operative autologous blood donation (PAD); and (2) pre-operative imaging, including questions about different types of imaging for different levels of curvature.

The operative section had three sub-sections: (1) operative technique, including two questions about the number of cross-links that surgeons use in a posterior construct; (2) operative graft use and implant assessment, including 27 questions about different types of grafts and implants; and (3) intra-operative monitoring and blood transfusion, including 10 questions about the use of intra-operative cell salvage, type of neurophysiology used, and the number of consultants present during surgery.

The infection control section included two questions about the use of drains during postoperative management and the infection control procedure(s) employed.

Data from the questionnaires was entered into Microsoft Excel for further analysis. The Excel data was coded into numbers for use with statistical package for social sciences (SPSS) software 16.0 (SPSS Inc., Chicago, IL, USA). Data was presented as mean and standard deviation ± standard deviation for continuous variables, with a confidence level of 95%. Frequencies and percentages were also used to present categorical variables. We also analyzed and compared the responses of the 18 orthopedic surgeons to those of the 18 neurosurgeons, using systematic random sampling. Chi-square test was used to test the association between the categorical variable, and a p value of < 0.05 was considered statistically significant.

## Results

**Figure 1:**
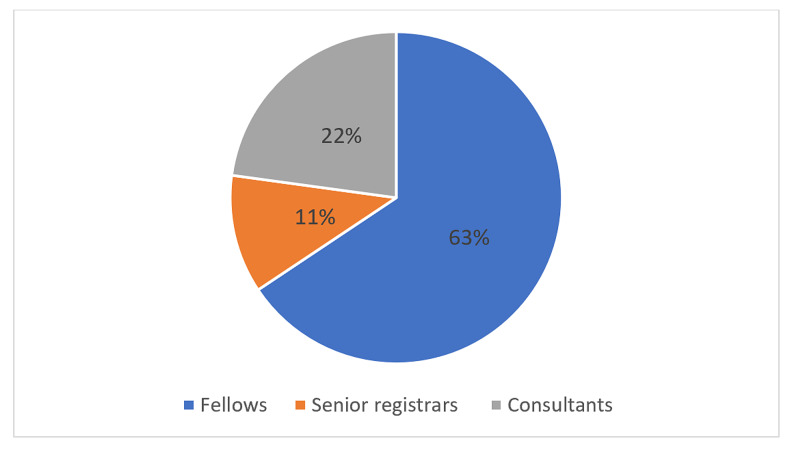
Participants’ current titles.

A total of 150 surgeons were included, of which 73 responded to the questionnaire (response rate of 48.6%). Of these, 46 (63.01%) were fellows, while 11 (15.07%) and 16 (21.92%) were senior registrars (associate consultant) and consultants, respectively (see Figure 1). All respondents were males, and 37 (51.39%) were aged between 30 and 40 years.

Fifty five (75.34%) of the participants were orthopedic surgeons, while 18 (24.33%) were neurosurgeons. Variations were observed regarding years of experience. Among all respondents, 29 (39.73%), 27 (36.99%), 11 (15.07%), and 6 (8.22%) had 1-5, 6-10, 11-15, and more than 15 years of experience, respectively. Of surgeons performing AIS operations, 37 (51.39%) reported less than 15 cases per year. The majority of respondents (51; 69.86%) were working in government hospitals, and these were predominantly located in the central region of Saudi Arabia (39; 53.42%).

**Figure 2:**
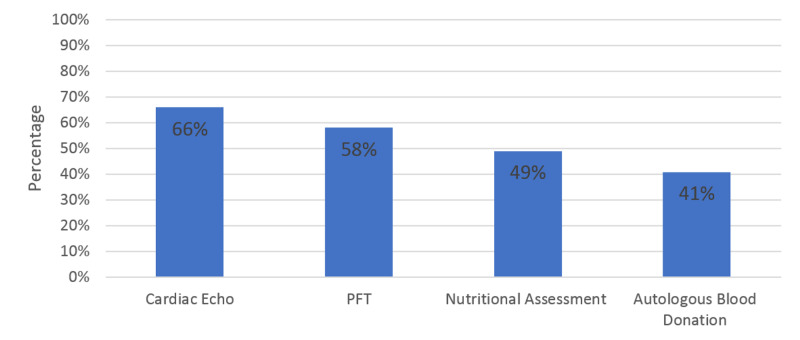
Pre-operative AIS patient’s assessment tests. AIS, adult idiopathic scoliosis; PFT, pulmonary function test.

Echocardiogram was the most commonly used pre-operative health assessment test, performed by 48 (66.67%) participants, followed by PFT and nutritional assessment, which were performed by 42 (58.33%) and 35 (49.3%), respectively. Moreover, 42 (59.15%) surgeons never performed autologous blood donation (see Figure 2). For pre-operative images assessment, whole spine MRI was always performed by 36 (52.94%) surgeons. Surgeons requested x-rays for curvatures between 40 degrees and 90 degrees as well as more than 90 degrees as follows: scoliosis series AP and lateral were requested by 66 (90%) and 65 (89%), respectively; lateral bending x-rays were requested by 57 (78%) and 58 (79.45%), respectively; and supine x-rays were requested by 14 (19%) and 18 (24.66%), respectively. Most surgeons (66; 90%) did not request traction x-rays for curvatures between 40 degrees and 90 degrees, while 34 (46.58%) requested these for curvatures greater than 90 degrees.

Regarding the posterior construct, 29 (45.31%) surgeons did not use cross-links for fewer than 10 levels, while 23 (35.94%) did not use cross-links for more than 10 levels. The most commonly used procedure in addition to local bone graft was allograft, which was employed by 37 surgeons (50.68%), while iliac crest autograft was used by only seven (10%). Likewise, 44 (68.75%) did not use bone morphogenetic protein.

A total of 55 (85.94%) participants favored an all-screw pedicle construct over a hybrid construct. Additionally, 26 (40.63%) used x-ray to assist with the screw insertion, while 26 (40.63%) used anatomical landmarks exclusively. Intra-operative cell salvage was not used by 26 surgeons (40.63%), while 28 (43.75%) employed it in certain cases.

Motor and sensory neurophysiological monitoring was employed by 47 (73.44%) participants. Intra-operative use of traction was used on a case-by-case basis by 29 (46.03%) participants. However, a similar number reported not using this method. A total of 47 (73.44%) stated that one consultant was present during surgery, and 32 (50%) reported that they inserted a surgical drain during the operation.

Results were compared for 18 neurosurgeons and 18 orthopedic surgeons. Statistically significant disagreements were found for pre-operative assessment. A whole spine MRI was always requested by 72% of neurosurgeons versus 44% of orthopedic surgeons, respectively. Meanwhile, 27% of orthopedic surgeons had never requested a whole spine MRI, and none of the neurosurgeons ignored requests for the test (p = 0.019 and 0.022, respectively).

For curvatures between 40 degrees and 90 degrees, bending x-ray was requested by 55% of neurosurgeons and 88% of orthopedic surgeons, respectively (p = 0.047) (see Table 1). A statistically significant association was found between the age of the surgeons and the type of graft used (p = 0.004). Surgeons aged between 30 and 40 years used local bone graft in addition to allograft more frequently than any other age group. The association between surgeons’ age and their use of x-ray for a typical curvature between 40 degrees and 90 degrees and curvature greater than 90 degrees was statistically insignificant (p = 0.868 and 0.067, respectively). Likewise, the association between the surgeon’s age and their infection control practices was statistically insignificant (p = 0.491). The association between surgeons’ experience and the types of x-rays used for a typical curvature greater than 90 degrees was statistically significant (p = 0.011). Surgeons between 1 and 5 years of experience used scoliosis series AP/lateral, supine x-ray, and bending x-ray more frequently than other groups. However, there was no statistically significant association between experience and the type of x-ray used for a typical curvature between 40 degrees and 90 degrees (p = 0.086). Likewise, no significant associations were seen between surgeons’ experience and the type of bone graft used and infection control practices (p = 0.310 and 0.857, respectively).

**Table 1:** Comparison of responses by neurosurgeons and orthopedic surgeons. Values are presented as the number of surgeons. Only statistically significant differences are mentioned, with a p value < 0.05 taken to be significant.

	Residency training program N of surgeons		Statistically significant p value
	Neurosurgery	Orthopedics	
Operative Assessment:			
Using cross-link in a posterior construct of fewer than 10 levels: None One Two More than two	3 2 7 1	8 1 7 0	- - - -
Using cross-link in a posterior construct of more than 10 levels: None One Two More than two	3 1 3 6	6 2 7 1	- - - .013
Operative (Graft and Implants) Assessment:			
Type of graft used: Local bone + iliac crest autograft Local bone + allograft Local bone + substitutes Local bone + demineralized bone matrix	2 3 8 5	0 14 5 7	- .000 - -
Use of bone morphogenetic protein: Yes No	3 10	6 10	- -
Favored type of constructs: All-screw pedicle construct Hybrid construct	13 0	14 2	- -
Technique for screw insertion used: C-Arm (x-ray) Navigation None (using anatomical land marks) Both C-arm and navigation	7 1 2 3	4 2 9 1	- - .024 -
Operative (Monitoring and Blood transfusions) Assessment:			
Use of intra-operative cell salvage (saver): All cases Not used Case by case	0 6 7	3 8 5	- - -
Type of neurophysiology used: Sensory-evoked potentials alone Motor-evoked potentials alone Combination of motor and sensory monitoring None	1 1 8 3	0 4 11 1	- - - -
Use of intra-operative traction during surgery: All cases Not used Case by case	0 6 7	1 8 6	- - -
Number of consultants during surgery: One Two Variable	6 6 1	14 1 1	.017 .013 -
Infection Control Measures:			
Use of drains during postoperative management for optimal care: Yes No	10 3	8 8	- -

## Discussion

The surgical management of AIS patients has seen many developments in recent years, including new surgical technologies, the use of bone substitutes, spinal cord monitoring, and blood conservation techniques. All of these add complexity to surgeons’ decision-making and preferences [12,14]. This study explored variations in the management of AIS among spine surgeons in Saudi Arabia. The results showed a wide divergence and heterogeneity among respondents.

Regarding the pre-operative health assessment of AIS patients, surgeons’ choices varied for all sections addressed by the questionnaire. Most of these variations were in relation to the performance of nutritional blood tests and PFT, which may be due to differences in the pre-operative assessment protocols adopted by local hospitals. A previous study reported similar results, with 50% of surgeons stating that they perform PFT optimally and 42% assessing the nutritional status of their patients [6]. These results are contrary to those of an UK study for which there was consensus among surgeons on pre-operative assessment [7].

While radiographs have been used in both research and clinical practice, there is no hard evidence confirming the need for these [15]. This study showed relative agreement in the use of pre-operative whole spine MRI for AIS patients, with most surgeons agreeing that it should be used either all the time or on a case-by-case basis. This finding was consistent with the results of two previous studies [6,9]. Moreover, surgeons in the present study showed a consensus on the use of x-rays, especially for scoliosis series AP/lateral imaging. These results were also in line with those of an earlier study [6]. It is worth noting that variations in radiographic pre-operative modalities may be due to logistics, costs, and local guidelines.

Evidence exists to support the use of cross-links to improve construct stability and stiffness [16,17]. However, this evidence is controversial, with some studies suggesting that cross-linking does not achieve these ends [18,19]. In the present study, surgeons’ opinions on the use of cross-linking varied extensively for both fewer and more than 10 levels posterior constructs. This may be due to both the aforementioned controversy and the guidelines in force. Similar results were reported in a study by Michael et al., where more than half of the surgeons agreed on the use of more than two cross-links in posterior constructs of more than 10 levels [7].

Local bone grafts in addition to iliac crest autograft are considered the gold standard for spine fusion. However, many side effects and complications have been reported [20]. The use of allograft, bone substitutes, and demineralized bone matrix is widely supported in the literature [21-23]. In this study, half of the surgeons had similar use of allograft. However, less than 10% of surgeons reported using iliac crest grafts. No similarities were found with other studies, where surgeons’ preferences vary with regard to the use of bone substitutes and iliac crest autografts [6,7,9].

Bone morphogenetic protein (BMP) is used with bone grafts to induce bone formation. As well as its efficacy in enhancing bone fusion rates, it is generally safe to use. However, some suspicion remains regarding heterotopic ossification [24-26]. Most respondents in this study reported that they do not use BMP. This may be due to its availability or associated treatment. Similar results were also reported in previous studies, with surgeons reporting low rates of BMP use [6,7,13]. The vast majority of participants in this study mentioned that they prefer the all-screw pedicle construct to a hybrid construct, in line with results from previous studies [6,7].

There was a consensus in the present study about the use of intra-operative cell salvage in the intra-operative management of AIS patients. This is consistent with previous studies in which intra-operative cell salvage or autologous blood transfusion drains have been used [6,7]. It is worth mentioning that the use of intra-operative cell salvage decreases the need for blood transfusion [27,28]. Additionally, a combination of motor and sensory monitoring was used by the surgeons in this study as the optimal method for intra-operative neurophysiology. Similar results were reported in a study carried out in the United Kingdom [7]. Spinal cord injury may happen during spine surgery, either due to cord ischemia or direct transgression. Neurophysiological monitoring is hence important, either via somatosensory cortical-evoked potentials or transcranial motor-evoked potentials. A combination of these two is recommended [29]. Surgeons in this study reported that one consultant was present during surgery, despite the fact that the recommendations require the presence of two [8].

A limitation of this study was the small number of participants and the low response rate. Likewise, the participating surgeons were not selected randomly, which may affect the generalizability of results.

## Conclusions

A large variation was observed in surgical practices in AIS operations, including the use of cross-links, intra-operative cell salvage, and the intra-operative use of traction. Use of cross-links for fewer than 10 levels was the predominant practice, along with the use of allografts in addition to local bone grafts. Disparity emerged in the use of intra-operative traction, as participants were nearly halved between using and not using this technique. The same variability applied to a lesser extent for pre-operative assessment practices, with echocardiography being the most commonly utilized test and MRI being ordered more frequently by neurosurgeons than orthopedic surgeons, while orthopedic surgeons requested x-rays more often. Therefore, it is recommended that surgical practice guidelines be developed to build consensus among surgeons regarding surgical practices in AIS operations. Additionally, a unified surgical training curriculum may be of use in achieving this goal.
